# Quantum Diffusion in the Lowest Landau Level of Disordered Graphene

**DOI:** 10.3390/nano12101675

**Published:** 2022-05-14

**Authors:** Andreas Sinner, Gregor Tkachov

**Affiliations:** 1 Institute of Physics, University of Opole, 45-052 Opole, Poland; 2 Institute of Physics, University of Augsburg, 86135 Augsburg, Germany

**Keywords:** low-dimensional semimetals, electronic transport in graphene, quantum hall effect

## Abstract

Electronic transport in the lowest Landau level of disordered graphene sheets placed in a homogeneous perpendicular magnetic field is a long-standing and cumbersome problem which defies a conclusive solution for several years. Because the modeled system lacks an intrinsic small parameter, the theoretical picture is infested with singularities and anomalies. We propose an analytical approach to the conductivity based on the analysis of the diffusive processes, and we calculate the density of states, the diffusion coefficient and the static conductivity. The obtained results are not only interesting from the purely theoretical point of view but have a practical significance as well, especially for the development of the novel high-precision calibration devices.

## 1. Introduction

Two-dimensional (2d) electronic systems in general and their transport properties in particular have been in the focus of intense research for several decades. In such systems, the effects due to quantum interference are strong and give rise to the interesting and rather unintuitive phenomena, as for instance various facets of the quantum Hall effect. Yet another effect on the transport that is supposed to be strong in 2d arises from the disorder which is always present in realistic materials. In conventional 2d electron systems, which are characterized by a parabolic and isomorphic spectrum, the presence of the disorder is widely believed to lead to the destructive interference of electronic quantum waves and consequently to the suppression of the electronic transport through the system on macroscopic scales. This phenomenon is usually called the Anderson localization of electronic wave functions, and it has received much of attention in the past [1,2,3,4]. This picture was challenged with the discovery of the unconventional behavior of electrons in the transition between Hall plateaux in quantum Hall systems. The experimental evidence from this observations points to the principal possibility for the existence of a metallic state in 2d systems under special conditions [5]. However, a real change of paradigm occured with the discovery of metallic states in graphene [6,7,8] and in a number of further low-dimensional systems, which is collectively known as the topological insulators [9,10,11,12,13,14,15,16]. A feature common to all these systems is the presence of the so-called nodes in the band structure and the linearity of the spectrum in the vicinity of these nodes. Despite being pristine 2d systems, they reveal a finite dc conductivity, which is very robust against disorder and thermal fluctuations.

The theoretical approach to the electronic transport of disordered electron gases is a rather formidable and cumbersome undertaking. Because the translational symmetry in the system is explicitly broken by the randomness, the usual methods of the theoretical analysis, which are mainly built around the duality between the position and momentum space representations and the special role of the Fourier transformation as the diagonalization tool for the quadratic Hamiltonians, no longer work. Therefore, the main idea behind every analytical approach to the macroscopic disordered systems is to reintroduce the translational invariance into the system by mapping the initial problem, which usually neglects the electron–electron and electron–phonon interactions from the outset, by a kind of an averaging operation on an effective interacting model in which the scattering of individual electrons on the randomly distributed potentials is approximated by the interaction operators expressed in terms of bilineals of second quantization operators. However, in practical terms, such an averaging procedure works well only under a weak disorder assumption, which guarantees a well-formed saddle-like shape of the free energy functional. In this case, the main effects caused by the disorder are taken into account by the summation of all contributions in partial diagrammatic channels [2,3,4,17,18,19,20,21,22,23,24,25,26,27,28,29,30].

In magnetic fields, the quantum mechanics of charge carriers with a linear spectrum specific for graphene is governed by an interplay of the intrinsic and magnetic-field-induced Berry curvatures. Several aspects of this physics remain widely untouched, though. For instance, relatively little is known about the role of disorder and its interplay with the magnetic field. The overall progress in this area has been slow because of the technical challenges, which are considerable even by the standards of the community [31,32,33,34,35]. A number of issues make the disordered electrons in the homogeneous perpendicular magnetic field look differently than the situation without a magnetic field. Due to the freedom of the gauge choice, the problem can be approached in a number of ways, which differ very much in details and in the outer appearance. The popular choice of the central gauge has the advantage that the solutions of the Schrödinger equation are states localized in the position space. Therefore, one can do computations in the position space in an exact manner.

The envisaged problem is notoriously difficult because the model lacks a small expansion parameter [36]. This inevitably leads to divergent expansion series. A powerful method developed to keep such divergences under control is the renormalization group. In the past, our understanding of the physics of disordered metals and semiconductors profited vastly from the various combinations of variational and perturbative techniques with the renormalization group, c.f. Refs. [1,2,3,25,26,27] and Refs. [37,38,39]. However, in the central gauge picture, there is no continuous variable to be sliced off by iterations in order to obtain the renormalization group equations. Of course, one can use a different gauge, which allows for a description in terms of states localized in one direction and propagating in the other. The price to pay is the loss of exactness, which is too costly to give up. In this paper, we develop a diagrammatic approach to the conductivity of the two-dimensional disordered electron gas in a strong magnetic field in a central gauge picture. While these series can still be wrapped up exactly for the single-particle propagators, as it was impressively demonstrated by Wegner in Ref. [32], additional technical issues make elusive every attempt of applying these techniques with the same success to the two-particles propagators. The available divergent series cannot be directly plugged into the Kubo formula without some not a priori obvious regularization or resummation. Hence, the usual way to approach the conductivity is via the Einstein relation and correspondingly via the notion of diffusion [40,41,42]. Because the corresponding statistical averages require normalization with respect to the vacuum fluctuations [43], this provides a tool of estimating the measurable quantities by means of some kind of analytical continuation [34,35,44,45].

To make our approach function, it relies on the information from the perturbative expansion. Therefore, we perform the exact computations of the perturbative series to the very high order. We identify the exact asymptotics of the two-particles propagator functions and approach the diffusion coefficient via the mean squared displacement using these asymptotics. It turns out that the behavior at longer time is dominated by the higher-order elements and tends toward a stationary state. On the sublaying time scales though, there is a large region with linear time dependence, which is characteristic of the diffusion. To approach this regime, we propose a self-consistent equation of motion for the mean squared displacement and extract the diffusion coefficient from there. With the obtained diffusion coefficient and density of states, we find via the Einstein relation a universal expression for the static conductivity in the lowest Landau level. All the system becomes metallic within a parameter window around the eigenvalues of the Hamiltonian of the clean system. With increasing disorder, this parameter window becomes broader. Numerically, the conductivity of disordered gapless and undoped graphene is very close to the experimentally established values.

The structure of this paper is as follows: In Section 2, we briefly discuss the main facts about the tight-binding Hamiltonian on the honeycomb lattice, its eigenvalues and eigenstates, and introduce the effective continuous model. In Section 3, we elaborate on the topological properties of the Hamiltonian and its eigenstates. In Section 4, we proceed with the consideration of the effective Hamiltonian, which describes the graphene in a strong external magnetic field and evaluates the single-particle propagator of the clean system in Section 5. In Section 6, we evaluate the Kubo–Greenwood formula for the dc conductivity of the clean gapless and chemically neutral graphene off and in an external magnetic field. In Section 7, we approach the single-particle propagator of the disordered system and discuss the Wegner’s exact solution and the exact density of states. In Section 8, we give our result for the two-particles propagator and for the mean squared displacement. Finally, in Section 9, we extract the diffusion coefficient from the equation of motion of the mean squared displacement and with that the static conductivity.

## 2. Tight-Binding and Effective Hamiltonian of Graphene

**Figure 1:**
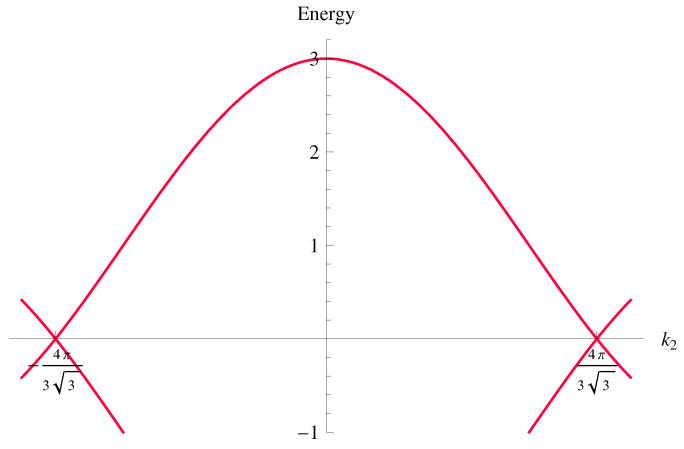
Spectrum of the tight-binding model along the line $k_{1}^{}=0$ with two Dirac cones at the corners of the Brilloun zone. The energy axis is scaled in units of the hopping parameter between nearest-neighbors *t*.

First, we briefly review the main spectral and topological properties of the tight-binding Hamiltonian on a honeycomb lattice. In second quantization, it reads

(1) $$H_{\mathrm{TB}}^{}=-t\sum_{\langle rr^{\prime }\rangle }\;\left(c_{r}^{†}d_{r^{\prime }}^{}+d_{r^{\prime }}^{†}c_{r}^{}\right)\;,$$

where *c* and *d* ($c^{†}$ and $d^{†}$) denote the annihilation (creation) operators acting on the lattice sites of each sublattice of the honeycomb lattice, respectively. The nearest neighbor positions are $a_{1}^{}=a\left(0,-1\right),\;a_{2,3}^{}=\frac{a}{2}\left(\pm \sqrt{3},1\right)$, where *a* denotes the lattice spacing. The tight-binding Hamiltonian Equation (1) is translationally invariant and is diagonalized by a Fourier transform, giving the eigenvalues $E_{\pm }^{}=\pm E=\pm \sqrt{h_{1}^{2}+h_{2}^{2}}$ and the respective eigenstates of the first-quantized Hamiltonian

(2) $$|v_{\pm }^{}\rangle =\pm \frac{1}{\sqrt{2}E}{\left[(h_{1}^{}-ih_{2}^{})\;,\pm E\right]}^{T},$$

with $h_{1}^{}=-t\sum_{i=1}^{3}\cos\left(a_{i}^{}\cdot k\right)$ and $h_{2}^{}=-t\sum_{i=1}^{3}\sin\left(a_{i}^{}\cdot k\right)$. The eigenvalues of the tight-binding Hamiltonian vanish at nodal points at the corners of the hexagonal Brillouin zone. Each of the corners contributes with the fraction 1/3 to the total number of cones, which therefore is 2. At chemical neutrality, i.e., for Fermi energy laying precisely at nodal points, there is no extended Fermi surface, and it became common to talk about Fermi points or semimetals. Close to the Fermi points, the fermion dispersion is linear and therefore describes massless Dirac particles, cf. Figure 1. The two Dirac cones are not exactly equivalent though, but they differ by a subtle notion of chirality. The states corresponding to each of two cones can be thought of as the chiral partners of each other. The total chirality of the tight-binding Hamiltonian is therefore zero. Being interested in the physics at low energies, it is usually sufficient to use the effective low-energy Hamiltonian

(3) $$H=\Delta_{0}^{}\Sigma_{03}^{}+\epsilon_{0}^{}\Sigma_{00}^{}-iv\left(D_{+}^{}\nabla_{-}^{}+D_{-}^{}\nabla_{+}^{}\right),$$

where $\nabla_{\pm }^{}=\partial_{x}^{}\pm i\partial_{y}^{}$. To describe the 4×4 matrix body of the Hamiltonian, it is useful to introduce the Dirac matrices $\Sigma_{ab}^{}=\sigma_{a}^{}⊗\sigma_{b}^{}$, a,b=0,1,2,3, with $\sigma_{a=1,2,3}^{}$ denoting the Pauli matrices in their usual representation and $\sigma_{a=0}^{}$ being the two-dimensional unity matrix. The first index refers to the valley and the second refers to the sublattice degree of freedom. With that, $D_{\pm }^{}=1/2\left[\Sigma_{01}^{}\pm i\Sigma_{02}^{}\right]$ follows. The band gap $\Delta_{0}^{}$ in pristine graphene is usually attributed to the spin–orbit coupling and has the size of roughly $10^{-3}$ meV [11], but it can also be considered as a free parameter available for fine tuning. Finally, the chemical potential $\epsilon_{0}^{}$ is an adjustable quantity.

## 3. Topological Chern Number

**Figure 2:**
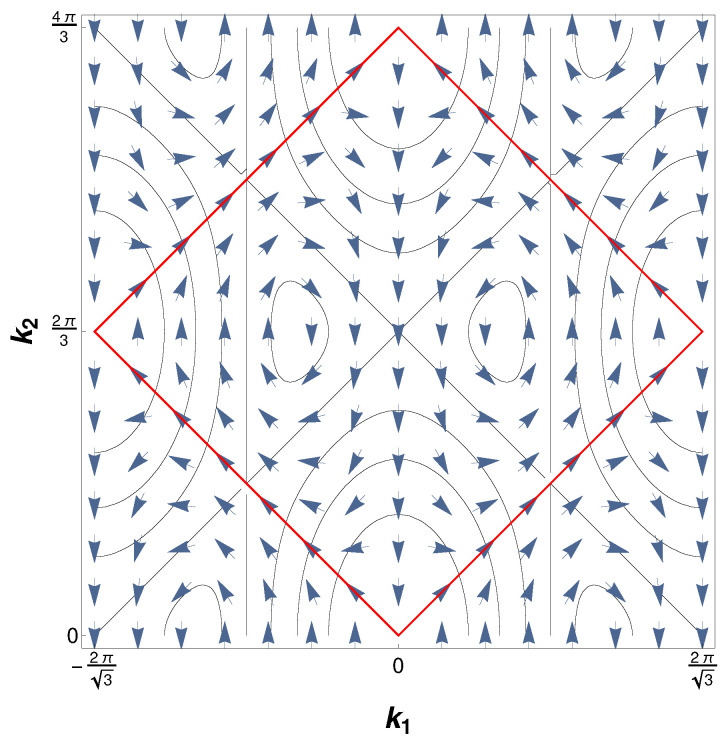
The circulation of the Berry vector potential corresponding to the occupied band of the full half filled tight-binding model in the reciprocal space with visible vortex-like structures around the position of the nodal points.

The phase of the wave function plays a crucial role for the properties of the related physical system. It is associated with a topological invariant called the Chern number and is ultimately responsible for the robust macroscopic properties, such as for instance the famous universal conductance. The Chern number is defined as a contour integral [46]

(4) $$C^{}=\frac{1}{2\pi }\oint_{C}d\vec{k}\cdot {\vec{A}}^{}\left(k\right)$$

over the so-called Berry vector potential $\vec{A}\left(k\right)=-i\langle v_{\pm }^{}|{\vec{\nabla }}_{k}^{}|v_{\pm }^{}\rangle$ along any closed path in the reciprocal space. Here, $|v_{\pm }^{}\rangle$ denotes an eigenstate of the Hamiltonian defined in Equation (2). The Berry vector potential corresponding to the completely filled band of the full tight-binding Hamiltonian is shown in Figure 2. It appears to have the shape of a double vortex centered around the nodal points of the spectrum and demonstrates nicely the difference in chirality of the Dirac cones by whirling in opposite directions. The total Chern appears as the sum of Chern numbers from each eigenstate. Therefore, the total Chern number of the pristine graphene is zero, but this might change if a fundamental symmetry of the Hamiltonian is broken, e.g., by applying a magnetic field.

## 4. The Effective Hamiltonian in Strong Magnetic Field

In strong magnetic fields, we replace the usual derivatives by the covariant ones $\partial_{\mu }^{}\to \partial_{\mu }^{}+iA_{\mu }^{}$, with the vector potential *A* related to the magnetic field via ∇×A=B. Here, we use the central gauge $A=B/2{\left(-y,x,0\right)}^{T}$, the choice which makes analytical calculations particularly convenient. Introducing complex coordinates z=x+iy, $\bar{z}=x-iy$, and corresponding derivatives $\partial_{z}^{}=\left(\partial_{x}^{}-i\partial_{y}^{}\right)/2$, $\partial_{\bar{z}}^{}=\left(\partial_{x}^{}+i\partial_{y}^{}\right)/2$, with the properties $\partial_{z}^{}z=\partial_{\bar{z}}^{}\bar{z}=1,\;\;\partial_{z}^{}\bar{z}=\partial_{\bar{z}}^{}z=0,$ we get $\nabla_{-}^{}\to 2\partial_{z}^{}+k^{2}\bar{z}=A$, $\nabla_{+}^{}\to 2\partial_{\bar{z}}^{}-k^{2}z=A^{†}$, where

(5) $$k^{2}=\frac{eB}{2\hbar }=\frac{1}{\ell^{2}}.$$

where ℓ=1/k is referred to as the magnetic length. The operator *A* annihilates the functions

(6) $$\varphi_{n}^{}\left(r\right)=\frac{k}{\sqrt{\pi }}\frac{{\left(k\bar{z}\right)}^{n}}{\sqrt{n!}}e^{-\frac{k^{2}}{2}z\bar{z}}$$

i.e., $A\varphi_{n}^{}\left(r\right)=0$, for every positive integer *n*. The Gaussian part of Equation (6) guarantees the localization in the position space and makes it possible to carry out an integration in the position space exactly. The holomorphic part of Equation (6) contains only powers of $\bar{z}$, and therefore, the wave function itself is manifestly chiral, which can be linked to the induced Berry curvature. The difference in the intrinsic Berry curvature discussed around Equation (4) is in the absence of the partner state with the opposite chirality, which reflects the explicit time-reversal symmetry breaking by an external magnetic field. The Hilbert space of the lowest Landau level is infinitely degenerate; i.e., *n* can assume every positive integer value between zero and infinity. In this notation, the Hamiltonian becomes

(7) $$H=\Delta_{0}^{}\Sigma_{03}^{}-\epsilon_{0}^{}\Sigma_{00}^{}-iv\left(D_{+}^{}A+D_{-}^{}A^{†}\right).$$

The ground state (i.e., the eigenstate in the lowest Landau level) suffices the condition

(8) $$iv\left(D_{+}^{}A+D_{-}^{}A^{†}\right)\psi =0,$$

which suggests two solutions:

(9) $$\psi_{+,n}^{}\left(r\right)=\varphi_{n}^{}\left(r\right)\left(\begin{array}{c} 0\\ 1\\ 0\\ 0 \end{array}\right),\;\;\;\psi_{-,n}^{}\left(r\right)=\varphi_{n}^{}\left(r\right)\left(\begin{array}{c} 0\\ 0\\ 1\\ 0 \end{array}\right),$$

which correspond to two valley polarization. The respective eigenvalues of the Hamiltonian for each spin projection in the lowest Landau level are found from the stationary Schrödinger equation

(10) Hψ=Eψ,

which yields for both spectral branches (or Landau sublevels) [47]

(11) $$\begin{array}{c} E_{\pm }^{}=-\epsilon_{0}^{}\pm \Delta_{0}^{}, \end{array}$$

i.e., the spectrum of both Dirac electron species consists of two flat bands irrespective of the strength of the magnetic field. Moreover, in chemically neutral and gapless graphene, the spectrum in the lowest Landau level is at zero [48].

## 5. Single-Particle Propagator in the Lowest Landau Level

The advanced (+) or retarded (−) Green’s function in the lowest Landau level can be calculated using the spectral representation

(12) $$\begin{array}{ccc} G_{r,r^{\prime }}^{\pm } & ∼ & \sum_{n=0}^{\infty }\varphi_{n}^{}\left(r\right){\bar{\varphi }}_{n}^{}\left(r^{\prime }\right)\sum_{s=\pm }\frac{P_{s}^{}}{E-E_{s}^{}\pm 0^{+}}, \end{array}$$

where $E_{s}^{}$ are the eigenvalues of the Hamiltonian for each spin projection in the lowest Landau level, as shown in Equation (11), and the normalization will be fixed later. The projectors $P_{\pm }^{}$ on the spin space

(13) $$P_{+}^{}=\left(\begin{array}{cccc} 0 & 0 & 0 & 0\\ 0 & 1 & 0 & 0\\ 0 & 0 & 0 & 0\\ 0 & 0 & 0 & 0 \end{array}\right)\;\;\;\mathrm{and}\;\;\;P_{-}^{}=\left(\begin{array}{cccc} 0 & 0 & 0 & 0\\ 0 & 0 & 0 & 0\\ 0 & 0 & 1 & 0\\ 0 & 0 & 0 & 0 \end{array}\right)$$

are idempotent and orthogonal matrices with properties $P_{+}^{}P_{-}^{}=0,\;\;P_{+}^{}P_{+}^{}=P_{+}^{},$$P_{-}^{}P_{-}^{}=P_{-}^{}$. The summation over all *n* yields

(14) $$\begin{array}{ccc} \sum_{n=0}^{\infty }\varphi_{n}^{}\left(r\right){\bar{\varphi }}_{n}^{}\left(r^{\prime }\right) & = & \frac{k^{2}}{\pi }e^{-\frac{k^{2}}{2}{\left(|z\right|}^{2}+|z^{\prime }{|}^{2})}\sum_{n=0}^{\infty }\frac{{\left(k^{2}\bar{z}z^{\prime }\right)}^{n}}{n!}=\frac{k^{2}}{\pi }e^{-\frac{k^{2}}{2}{\left(|z\right|}^{2}+|z^{\prime }{|}^{2}-2\bar{z}z^{\prime })}, \end{array}$$

which then gives for the Green’s function [32,49]

(15) $$G_{r,r^{\prime }}^{\pm }\left(E\right)=\frac{k^{2}}{2\pi }e^{-\frac{k^{2}}{2}{\left(|z\right|}^{2}+|z^{\prime }{|}^{2}-2\bar{z}z^{\prime })}\sum_{s=\pm }\frac{P_{s}^{}}{E-E_{s}^{}\pm 0^{+}}.$$

Notably, the local Green’s function ($r=r^{\prime }$) is a coordinate independent constant. The propagator is normalized this way in order to satisfy the usual sum rule

(16) ∓∫−∞∞dEπImtrGr,r±(E)=k2π=eBh,

where the trace operator acts only on the spin space. Equation (16) gives the number of the elementary flux quanta $\phi_{0}^{}=h/e$ per unit volume. In the real-time representation, the Green’s function represents a simple collection of undamped harmonic functions with the period determined by the eigenenergies of the lowest Landau level modes

(17) $$G_{r,r^{\prime }}^{\pm }\left(t\right)=\mp i\frac{k^{2}}{2\pi }e^{-\frac{k^{2}}{2}{\left(|z\right|}^{2}+|z^{\prime }{|}^{2}-2\bar{z}z^{\prime })}\sum_{s=\pm }P_{s}^{}e^{\pm iE_{s}^{}t},$$

and the initial time is assumed to be at zero. The Green’s function is totally separable on the space-time.

For the case of chemically neutral gapless graphene, the Green’s function becomes particularly simple [49]:

(18) $$G_{rr^{\prime }}^{\pm }\left(E\right)=\frac{k^{2}}{2\pi }\frac{1}{E\pm i0^{+}}e^{-\frac{k^{2}}{2}{\left(|z\right|}^{2}+|z^{\prime }{|}^{2}-2\bar{z}z^{\prime })}\left[P_{+}^{}+P_{-}^{}\right],$$

i.e., in the real-time representation, it is just a step function θ(t).

## 6. Static Conductivity of the Pristine Graphene vs. the Lowest Landau Level

The static conductivity of the clean system can be evaluated from the Kubo–Greenwood formula [28,29,34,35,50]:

(19) $$\sigma_{\mu \mu }^{dc}=\frac{e^{2}}{h}\lim_{E\to 0}E^{2}\;\mathrm{tr}\int d^{2}r\;r_{\mu }^{2}\;G_{r,0}^{+}\left(E\right)G_{0,r}^{-}\left(E\right).$$

We first evaluate this expression for the pristine graphene without a magnetic field. The Green’s function of such system reads

(20) $$G_{r,r^{\prime }}^{\pm }\left(E\right)=\int \frac{d^{2}q}{{\left(2\pi \right)}^{2}}\;e^{-iq\cdot (r-r^{\prime })}{\left[\pm iE\Sigma_{00}^{}+q\cdot J\right]}^{-1}=\int \frac{d^{2}q}{{\left(2\pi \right)}^{2}}\;e^{-iq\cdot (r-r^{\prime })}G^{\pm }\left(q\right),$$

where

(21) $$J_{\mu }^{}=\frac{\partial H}{\partial q_{\mu }^{}}$$

denotes the current operator, while the second power of the position operator can be written as

(22) $$r_{\mu }^{2}=-{\left.\frac{\partial^{2}}{\partial q_{\mu }^{2}}\right|}_{q=0}e^{-iq\cdot r}.$$

Therefore, the Kubo–Greenwood formula changes to

(23) $$\sigma_{\mu \mu }^{dc}=\frac{e^{2}}{h}\lim_{E\to 0}E^{2}\;\mathrm{tr}\int \frac{d^{2}q}{{\left(2\pi \right)}^{2}}\;J_{\mu }^{}G_{q}^{-}\left(E\right)G_{q}^{+}\left(E\right)J_{\mu }^{}G_{q}^{+}\left(E\right)G_{q}^{-}\left(E\right).$$

Taking into account

(24) $$G_{q}^{\pm }\left(E\right)G_{q}^{\mp }\left(E\right)=\frac{1}{q^{2}+E^{2}},$$

we then get to

(25) $$\sigma_{\mu \mu }^{dc}=\frac{e^{2}}{h}\lim_{E\to 0}\int \frac{d^{2}q}{{\left(2\pi \right)}^{2}}\;\frac{4E^{2}}{{\left[q^{2}+E^{2}\right]}^{2}},$$

with 4 being the trace of the unity matrix. Assuming an infinitely large upper cutoff, we finally get for the conductivity a universal number

(26) $$\sigma_{\mu \mu }^{dc}=\frac{1}{\pi }\frac{e^{2}}{h},$$

which is the famous universal conductivity of graphene [6]. Remarkably, this result is also valid for the case of weakly disordered Dirac electron gas [30,51,52,53].

For the calculation of the static conductivity in the lowest Landau level of clean gapless and undoped graphene, we employ the Green’s function shown in Equation (18). Here, we can evaluate the Kubo–Greenwood formula directly in the position space

(27) $$\begin{array}{ccc} \sigma_{\mu \mu }^{dc} & = & \frac{e^{2}}{h}{\left(\frac{k^{2}}{2\pi }\right)}^{2}\lim_{E\to 0}E^{2}\int d^{2}r\;r_{\mu }^{2}e^{-k^{2}r^{2}}\frac{2}{E^{2}}=\frac{1}{4\pi }\frac{e^{2}}{h}, \end{array}$$

where 2 is the trace of the matrix $P_{+}^{}+P_{-}^{}$. In addition, here is the conductivity of a universal number, but its magnitude is only a quarter of the clean graphene. It is obvious that this result is solely due to the presence of the zero mode in the spectrum of the gapless and chemically neutral graphene. A slightest doping or a smallest spectral gap would destroy this dc conductivity. Because of this fragility, we can think of the resulting Equation (27) as an anomaly in the parametric space of infinitely small thickness. In analogy to the situation without a magnetic field, we expect the widening of this line by disorder [53].

## 7. Single-Particle Propagator Renormalization Due to the Disorder

The disorder is introduced in the form of the fluctuating chemical potential v(r), which couples in the spin space to the unity matrix $\Sigma_{00}^{}$, with the white noise correlator:

(28) $${\langle v_{r}^{}\rangle }_{g}^{}=0,\;\;{\langle v_{r_{1}^{}}^{}v_{r_{2}^{}}^{}\rangle }_{g}^{}=g\delta_{r_{1}^{}r_{2}^{}}^{}.$$

The averaged propagator reads

(29) $${\bar{G}}_{r_{1}^{}r_{2}^{}}^{\pm }={\langle {\left[{\left(G^{\pm }\right)}^{-1}+v\Sigma_{00}^{}\right]}_{r_{1}^{}r_{2}^{}}^{-1}\rangle }_{g}^{}.$$

To perform the disorder average perturbative, we expand the propagator in powers of *v*. Because of the properties of the disorder correlator Equation (28), all terms with an odd number of potentials *v* vanish. The series then becomes

(30) $$\begin{array}{ccc} & {\bar{G}}_{r_{1}^{}r_{2}^{}}^{\pm }=\langle G_{r_{1}^{}r_{2}^{}}^{\pm }+G_{r_{1}^{}x_{1}^{}}^{\pm }v_{x_{1}^{}}^{}G_{x_{1}^{}x_{2}^{}}^{\pm }v_{x_{2}^{}}^{}G_{x_{2}^{}r_{2}^{}}^{\pm }+G_{r_{1}^{}x_{1}^{}}^{\pm }v_{x_{1}^{}}^{}G_{x_{1}^{}x_{2}^{}}^{\pm }v_{x_{2}^{}}^{}G_{x_{2}^{}x_{3}^{}}^{\pm }v_{x_{3}^{}}^{}G_{x_{3}^{}x_{4}^{}}^{\pm }v_{x_{4}^{}}^{}G_{x_{4}^{}r_{2}^{}}^{\pm } & \\ & +G_{r_{1}^{}x_{1}^{}}^{\pm }v_{x_{1}^{}}^{}G_{x_{1}^{}x_{2}^{}}^{\pm }v_{x_{2}^{}}^{}G_{x_{2}^{}x_{3}^{}}^{\pm }v_{x_{3}^{}}^{}G_{x_{3}^{}x_{4}^{}}^{\pm }v_{x_{4}^{}}^{}G_{x_{4}^{}x_{5}^{}}^{\pm }v_{x_{5}^{}}^{}G_{x_{5}^{}x_{6}^{}}^{\pm }v_{x_{6}^{}}^{}G_{x_{6}^{}r_{2}^{}}^{\pm }{\cdots \rangle }_{g}^{}. \end{array}$$

Here, the summation over repeating indices is understood.

The Green’s function shown in Equation (15) is spanned by the spin projectors $P_{s}^{}$. Therefore, only the disorder diagonal in the spin space is of importance. In addition to the randomly fluctuating chemical potential considered here, these might include the randomly fluctuating gap, which couples to $\Sigma_{03}^{}$, the random “chiral” chemical potential ($\Sigma_{30}^{}$), or the random “chiral” mass ($\Sigma_{33}^{}$). Each product of these matrices with $P_{s}^{}$ projects them bar the sign back onto $P_{s}^{}$ again. Therefore, the perturbative series shown in Equation (30) does not depend on a particular disorder type, and our analysis is generic and disorder type independent.

The exact Green’s function of disordered electrons in the lowest Landau level was obtained by Wegner in Ref. [32] in the distinctly separable form

(31) $${\bar{G}}_{rr^{\prime }}^{\pm }\left(E\right)=\frac{k^{2}}{\pi }e^{-\frac{k^{2}}{2}{\left(|r\right|}^{2}+|r^{\prime }{|}^{2}-2\bar{r}r^{\prime })}\sum_{s=\pm }F_{s}^{\pm }\left(E\right)P_{s}^{}.$$

**Figure 3:**
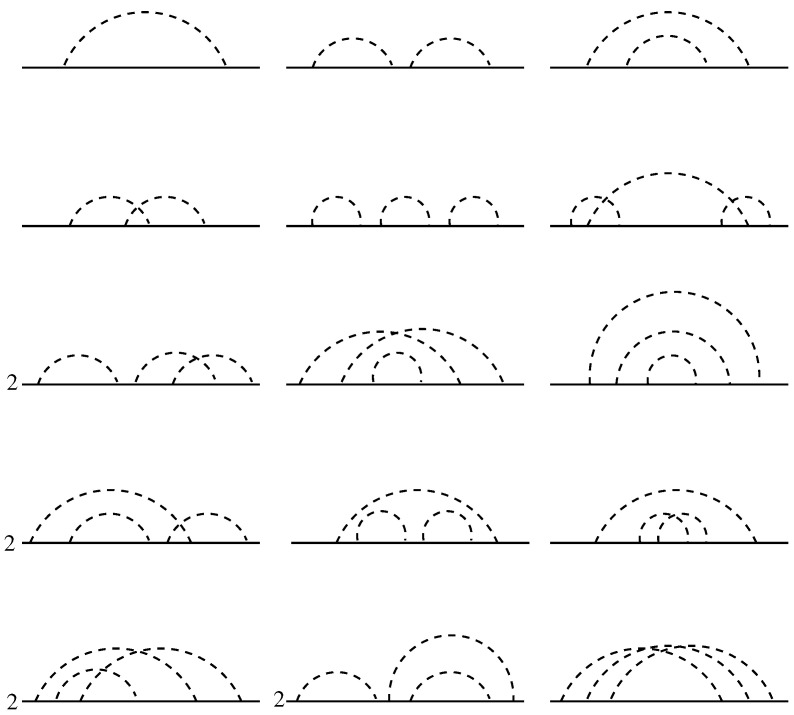
Perturbative processes contributing to the dressing of the single-particle propagator due to the disorder to order $g^{1}$ (one diagram), $g^{2}$ (three diagrams), and $g^{3}$ (fifteen diagrams). Some of the diagrams of order $g^{3}$ should be counted twice because of the degeneracy due to the mirror symmetry with respect to the imaginable vertical axis, which is accounted for by the factors 2 in front of them.

The frequency-dependent part of the Green’s function evaluated to the order $g^{3}$ by evaluation of the diagrams shown in Figure 3 reads

(32) $$\begin{array}{ccc} F_{s}^{\pm }\left(E\right) & = & \frac{1}{2}\frac{1}{E-E_{s}^{}}\left[1+\frac{E_{g}^{2}}{{\left[E-E_{s}^{}\right]}^{2}}+\frac{5}{2}\frac{E_{g}^{4}}{{\left[E-E_{s}^{}\right]}^{4}}+\frac{37}{4}\frac{E_{g}^{6}}{{\left[E-E_{s}^{}\right]}^{6}}\cdots \right], \end{array}$$

where $E_{g}^{2}=\frac{gk^{2}}{4\pi }$. The expansion coefficients 1, 1, 5/2, 37/4... are precisely those of the Wegner’s exact solution [32]. They are determined as expansion coefficients of the function

(33) $$-\frac{\partial }{\partial a}\log\left[\frac{2\pi }{\sqrt{b}}e^{-\frac{a^{2}}{b}}\int_{\frac{a}{\sqrt{b}}}^{\infty }dt\;e^{-t^{2}}\right].$$

in powers of $b/a^{2}$. Following [32], we find for the frequency-dependent part of the dressed single-particle propagator

(34) $$F_{s}^{\pm }\left(E\right)=\eta_{s}^{}\left(E\right)\mp i\rho_{s}^{}\left(E\right),$$

with the following explicit expressions for the real

(35) $$\eta_{s}^{}\left(E\right)=\frac{1}{E_{g}^{}}\left(\frac{2}{\pi }\frac{e^{\nu_{s}^{2}}\int_{0}^{\nu_{s}^{}}dt\;e^{t^{2}}}{1+{\left(\frac{2}{\sqrt{\pi }}\int_{0}^{\nu_{s}^{}}dt\;e^{t^{2}}\right)}^{2}}-\nu_{s}^{}\right),$$

and imaginary parts [33,34]

(36) $$\rho_{s}^{}\left(E\right)=\frac{1}{\sqrt{\pi }E_{g}^{}}\frac{e^{\nu_{s}^{2}}}{1+{\left(\frac{2}{\sqrt{\pi }}\int_{0}^{\nu_{s}^{}}dt\;e^{t^{2}}\right)}^{2}}.$$

They depend on the dimensionless energy

(37) $$\nu_{s}^{}=\frac{E-E_{s}^{}}{E_{g}^{}},\;\;\;\mathrm{where}\;\;E_{g}^{2}=\frac{gk^{2}}{4\pi }.$$

In the chosen units, the disorder-related energy $E_{g}^{}$ is a dimensionless quantity, which is proportional to the ratio of two relevant lengths $E_{g}^{}∼l_{\lambda }^{}/\ell$: the magnetic length ℓ∼1/k and the disorder related length $l_{\lambda }^{}∼\sqrt{g}$. The total density of states

**Figure 4:**
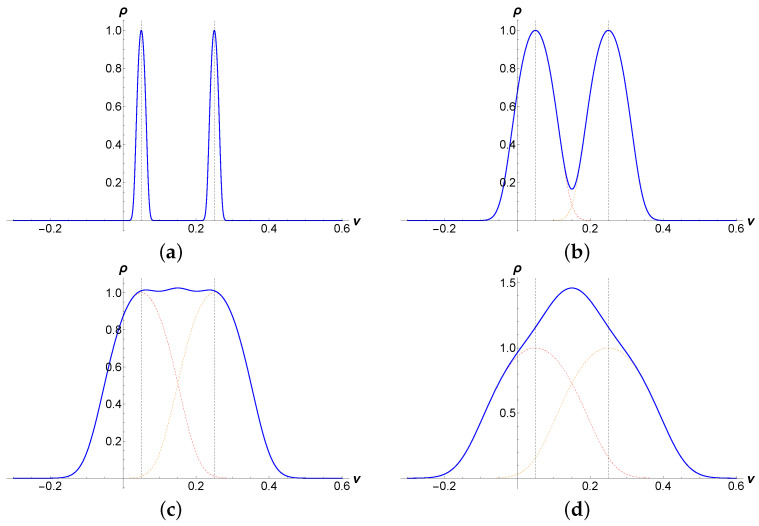
Evolution of the DOS of both Landau sublevels defined in Equation (11) (**a**–**d**) plotted in units of the DOS at each suband center $\frac{1}{\pi^{5/2}}\frac{k^{2}}{E_{g}^{}}$ with increasing disorder strength as a function of the dimensionless energy ν. The following quantities are used: $\epsilon_{0}^{}/t=0.15$, $\Delta_{0}^{}/t=0.1$ and $E_{g}^{}/t=0.01,0.045,0.073,$ and 0.1 in units of the hopping amplitude. Dashed lines emphasize the position of each eigenvalue.

(38) ρ(E)=∓1πImtrGrr±(E)=1π5/2k2Eg∑s=±eνs21+2π∫0νsdtet22.

is correctly normalized in accordance with Equation (16). Figure 4 shows the DOS from Equation (38). For weak disorder strength, the density of states that appears has the form of two sharp peaks placed symmetrically around the energy eigenvalues in the lowest Landau level. It is plotted in units of $\frac{1}{\pi^{5/2}}\frac{k^{2}}{E_{g}^{}}∼{\left(\ell l_{\lambda }^{}\right)}^{-1}$, $\ell l_{\lambda }^{}$ being the parametric volume constructed from the two specific lengths of the model. The peaks become broader with increasing disorder strength and overlap with each other until they merge to a single structure.

For the gapless and chemically neutral graphene, both peaks overlap and form a unique structure around the zero energy

(39) $$\rho \left(E\right)=\frac{2}{\pi^{5/2}}\frac{k^{2}}{E_{g}^{}}\frac{e^{\nu^{2}}}{1+{\left(\frac{2}{\sqrt{\pi }}\int_{0}^{\nu }dt\;e^{t^{2}}\right)}^{2}},\;\;\nu =\frac{E}{E_{g}^{}}.$$

Therefore, at the band center, we get

(40) $$\rho \left(0\right)=\frac{2}{\pi^{5/2}}\frac{k^{2}}{E_{g}^{}}.$$

## 8. Mean Squared Displacement of the Disordered System

The access to the diffusion goes via the mean squared displacement

(41) $$\langle r_{\mu }^{2}\left(t\right)\rangle =\frac{\mathrm{tr}\sum_{r}r_{\mu }^{2}P_{r0}^{}\left(t\right)}{\mathrm{tr}\sum_{r}P_{r0}^{}\left(t\right)},$$

where $r_{\mu }^{}$ is the position operator and $P_{rr^{\prime }}^{}\left(t\right)$ is the return probability density defined as

(42) $$P_{rr^{\prime }}^{}\left(t\right)=\int \frac{dE}{2\pi }\;e^{-iEt}P_{rr^{\prime }}^{}\left(E\right),$$

where

(43) $$P_{rr^{\prime }}^{}\left(E\right)={\langle G_{rr^{\prime }}^{+}\left(E\right)G_{r^{\prime }r}^{-}\left(E\right)\rangle }_{g}^{},$$

is the disorder averaged two-particles propagator. Notably, the numerator of Equation (41) appears to be essentially the Kubo–Greenwood formula shown in Equation (19). The relation between the mean squared displacement and diffusion is established via

(44) $${\left.\frac{d}{dt}\right|}_{t=0}\langle r_{\mu }^{2}\left(t\right)\rangle =2D,$$

where *D* is the diffusion coefficient. If we would be able to determine the diffusion coefficient of the disordered system through the direct evaluation of Equation (41), then it will be possible to compute the conductivity from the Einstein relation (in this particular form adopted from [29,50])

(45) $$\sigma =\frac{e^{2}}{\hbar }D\rho \left(E\right),$$

where ρ(E) is the density of states discussed in the previous paragraph.

**Figure 5:**
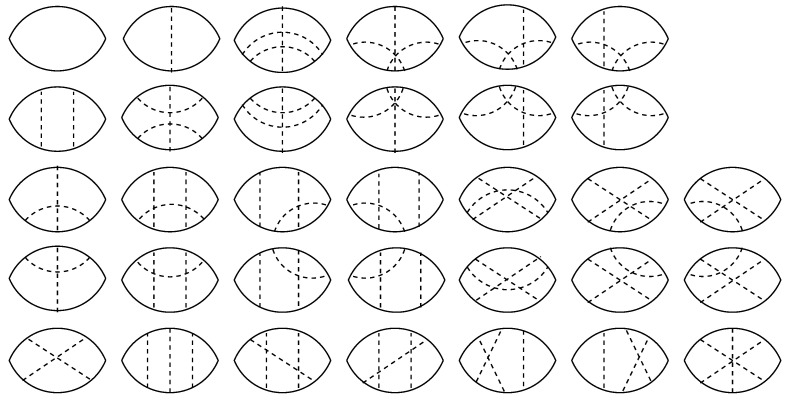
Perturbative processes contributing to the dressing of the two-particles propagator up to the third order in disorder strength. Solid lines denote the fully dressed Wegner’s propagators and the dashed lines denote the disorder correlators.

A rigorous evaluation of the full perturbative series for the two-particles propagator ${\langle G_{r,0}^{+}G_{0,r}^{-}\rangle }_{g}^{}$ along the lines of Wegner’s calculations for the single-particle propagator is principally impossible. Therefore, we need to consider the spatial averages. We evaluate both expressions from the numerator and denominator of Equation (41) perturbatively. Evaluation of all perturbative diagrams to order $g^{3}$, shown in Figure 5 yields for the expression in the numerator of Equation (41)

(46) $$\begin{array}{c} \mathrm{tr}\sum_{r}r_{\mu }^{2}P_{r0}^{}\left(E\right)=\frac{1}{4\pi }\frac{1}{E_{g}^{2}}\sum_{s=\pm }^{}{\left(2X_{s}^{}\right)}^{2}\left[\frac{1}{2}+{\left(2X_{s}^{}\right)}^{2}+2{\left(2X_{s}^{}\right)}^{4}+\frac{167}{36}{\left(2X_{s}^{}\right)}^{6}+\cdots \right.\\ \left.\left(\frac{3}{4}{\left(2X_{s}^{}\right)}^{4}+\frac{343}{72}{\left(2X_{s}^{}\right)}^{6}+\cdots \right)\cos2\phi_{s}^{}+\left(\frac{139}{72}{\left(2X_{s}^{}\right)}^{6}+\cdots \right)\cos4\phi_{s}^{}+\cdots \right], \end{array}$$

where

(47) $$X_{s}^{2}\left(E\right)=E_{g}^{2}\left[\eta_{s}^{2}\left(E\right)+\rho_{s}^{2}\left(E\right)\right]\;\;\mathrm{and}\;\;\phi_{s}^{}\left(E\right)=\arctan\left[\frac{\rho_{s}^{}\left(E\right)}{\eta_{s}^{}\left(E\right)}\right].$$

According to Equations (35) and (36), $X_{s}^{2}\left(E\right)$ and $\phi_{s}^{}\left(E\right)$ are dimensionless functions of the argument $\nu_{s}^{}=\left(E-E_{s}^{}\right)/E_{g}^{}$. The analogous computation for the denominator of Equation (41) yields

(48) $$\begin{array}{c} \mathrm{tr}\sum_{r}P_{r0}^{}\left(E\right)=\frac{k^{2}}{4\pi }\frac{1}{E_{g}^{2}}\sum_{s=\pm }^{}{\left(2X_{s}^{}\right)}^{2}\left[1+{\left(2X_{s}^{}\right)}^{2}+\frac{3}{2}{\left(2X_{s}^{}\right)}^{4}+\frac{13}{4}{\left(2X_{s}^{}\right)}^{6}+\cdots \right.\\ \left.\left({\left(2X_{s}^{}\right)}^{4}+\frac{9}{2}{\left(2X_{s}^{}\right)}^{6}+\cdots \right)\cos2\phi_{s}^{}+\left(\frac{5}{2}{\left(2X_{s}^{}\right)}^{6}+\cdots \right)\cos4\phi_{s}^{}+\cdots \right]. \end{array}$$

A reasonable approximation for the two-particles propagator that leads beyond this partially rigorous result includes all diagrams of the so-called ladder channel. The four lowest order ladder diagrams are evaluated as [54]

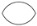
$=\frac{1}{4E_{g}^{2}}{\left(\frac{k^{2}}{\pi }\right)}^{2}\sum_{s=\pm }{\left(2X_{s}^{}\right)}^{2}\exp\left[-k^{2}r^{2}\right],$   (49)
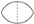
$=\frac{1}{4E_{g}^{2}}{\left(\frac{k^{2}}{\pi }\right)}^{2}\sum_{s=\pm }\;\frac{{\left(2X_{s}^{}\right)}^{4}}{2}\exp\left[-\frac{k^{2}r^{2}}{2}\right],$   (50)
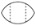
$=\frac{1}{4E_{g}^{2}}{\left(\frac{k^{2}}{\pi }\right)}^{2}\sum_{s=\pm }\;\frac{{\left(2X_{s}^{}\right)}^{6}}{3}\exp\left[-\frac{k^{2}r^{2}}{3}\right],$   (51)
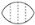
$=\frac{1}{4E_{g}^{2}}{\left(\frac{k^{2}}{\pi }\right)}^{2}\sum_{s=\pm }\;\frac{{\left(2X_{s}^{}\right)}^{8}}{4}\exp\left[-\frac{k^{2}r^{2}}{4}\right],$   (52)
which suggests the following asymptotics of the two-particles propagator in the form of an infinite series:

(53) $$P_{r0}^{\mathrm{lad}}\left(E\right)\approx \frac{1}{4E_{g}^{2}}{\left(\frac{k^{2}}{\pi }\right)}^{2}\sum_{s=\pm }\;\sum_{n=1}^{\infty }\frac{{\left(2X_{s}^{}\right)}^{2n}}{n}\exp\left[-\frac{k^{2}r^{2}}{n}\right].$$

Using this expression, one can complement Equations (46) and (48) to any order.

## 9. Equation of Motion for the Mean Squared Displacement

The equation of motion for the mean squared displacement has the form of the second-order ordinary differential equation

(54) $$k^{2}\frac{\partial^{2}}{\partial t^{2}}\langle r_{\mu }^{2}\left(t\right)\rangle =-E_{g}^{2}I_{N}^{}\left[\frac{N}{2}-k^{2}\langle r_{\mu }^{2}\left(t\right)\rangle \right],$$

where *N* refers to the order of the perturbative expansion. The expression for the integral $I_{N}^{}$ can be found in [54]. For our purposes, it is only important that it decreases with increasing *N*.

The large-time asymptotics ($t\gg t_{c}^{}$, $t_{c}^{}$ being some crossover time far in the past, which can be chosen zero) is given by the solution of the self-consistent equation

(55) $${\langle r_{\mu }^{2}\left(t\right)\rangle }^{>}\approx \frac{N}{2}\ell^{2}+\ell^{2}C\exp\left[-\frac{t}{\tau }\right],$$

where we introduced the scattering time as

(56) $$\tau \approx \frac{1}{\sqrt{I_{N}^{}}E_{g}^{}}.$$

In the limit t→∞, the mean squared displacement approaches its upper bound

(57) $$\lim_{t\to \infty }{\langle r_{\mu }^{2}\left(t\right)\rangle }^{>}\to \frac{N}{2}\ell^{2},$$

which for N→∞ lies in the infinity and is therefore never reached. Hence, it can be only approached from below, which requires C to be negative. If $\sqrt{I_{N}^{}}$ is small, then τ is large, and the regime with linear time dependence should be broad. The diffusion coefficient is then obtained from

(58) $${\left.\frac{\partial }{\partial t}{\langle r_{\mu }^{2}\left(t\right)\rangle }^{>}\right|}_{t=t_{c}^{}}=\left|C\right|\frac{\ell^{2}}{\tau }.$$

Formally, |C| should follow from the initial condition at $t=t_{c}^{}$, but for this, we need to know ${\langle r_{\mu }^{2}\left(t_{c}^{}\right)\rangle }^{>}$, which lies far in the past and is therefore forgotten. In order to be a physical quantity, we demand for *D* an invariance with respect to *N*. This is similar to the version of the renormalization group typically used in the high-energy physics. This implies

(59) $$\frac{\partial }{\partial N}(\sqrt{I_{N}^{}}\left|C|\right)=0,$$

from where then follows

(60) $$\sqrt{I_{N}^{}}\left|C\right|=\mathrm{const}.$$

Even though this constrain might appear not entirely transparent, it has a natural analogy in the case of disordered electron gas without a magnetic field. Here, the diffusion coefficient is determined from the self-consistent Born approximation and appears unchanged in the partial series, e.g., cooperon or diffuson [22,23]. A comparison with Equation (44) suggests this constant to be 2. Then, the physical diffusion coefficient becomes

(61) $$D\approx \frac{E_{g}^{}}{k^{2}}∼\ell l_{\lambda }^{},$$

i.e., it is proportional to the parametric volume of the model.

Inserting the density of states from Equation (38) and the diffusion coefficient Equation (61) into the Einstein relation Equation (45) yields the conductivity. The system is conducting within a parametric window located around each of the Landau sublevels. The width of the conducting window is determined by the parameters of the microscopic model and by the disorder. The transition g→0 is smooth, and the conductivity degenerates to two sharp peaks at the Landau sublevels. With increasing disorder, the peaks become broader and merge at some point to an amorphous structure. Simultaneously, the amplitude becomes smaller, signaling the suppression of the conductivity in the strong disorder limit.

We are now in the position to compute the conductivity at an arbitrary Landau sublevel defined in Equation (11). The corresponding density of the states is shown in Figure 4. For weak disorder, the contribution from the other sublevel is negligible, and we get for the density of states

(62) $$\rho_{\mathrm{sl}}^{}\left(0\right)\approx \frac{1}{\pi^{5/2}}\frac{k^{2}}{E_{g}^{}}.$$

Using the units of $e^{2}/h$ instead of $e^{2}/\hbar$ adds an extra factor of 2π, i.e.,

(63) $$\sigma_{\mathrm{sl}}^{}=2\pi D\rho_{\mathrm{SL}}^{}\left(0\right)\frac{e^{2}}{h}\approx \frac{2\pi }{\pi^{5/2}}\frac{e^{2}}{h}\approx 0.36\frac{e^{2}}{h}.$$

Numerically, it is close to the dc conductivity in clean graphene off the magnetic field $\frac{e^{2}}{\pi h}\approx 0.32\frac{e^{2}}{h}$ evaluated in Equation (26), which is valid even for the weakly disordered systems [30,51,52,53]. For us, the most interesting limit is the case of the neutral and gapless graphene, for which a rich empirical knowledge is available. Our estimation would give for this zero mode

(64) $$\sigma_{\mathrm{zm}}^{}\left(0\right)=2\sigma_{\mathrm{sl}}^{}\approx 0.72\frac{e^{2}}{h}.$$

Respected experimental studies of Refs. [55,56] determine the room-temperature longitudinal resistivity at the band center in the lowest Landau level as roughly 35 kΩ and 42 kΩ, respectively, which corresponds to (with $h/e^{2}\approx 25812\;\Omega$) to

(65) $$\sigma_{\exp}^{}\left(0\right)\approx 0.614\frac{e^{2}}{h}÷0.737\frac{e^{2}}{h},$$

which is surprisingly close to our estimation. The comparison is justified, since the disorder can be considered as an effective temperature, cf. [57] and references therein.

## 10. Discussions

The diffusion of electrons in random environments confined to the lowest Landau level in two spatial dimensions is a long-standing and conceptually challenging problem of quantum statistical mechanics. Without disorder, the quantum mechanical description of the problem is simply that of the harmonic oscillator with a discrete, though highly degenerate spectrum, comprising of the so-called Landau levels. In the strong magnetic field, the gap between the lowest and the first Landau levels is very large and only the lowest Landau level is relevant. In this regime, the electrons are distributed between the stationary Landau orbits in the position space and should stay there forever, thus forbidding any transport across the sample. This is due to the disorder that the electrons can move from one Landau orbit to another, producing an observable current. While several analytical approaches have been developed in the past for systems of disorder electrons off a magnetic field, a meaningful formulation of the problem in the strong magnetic field is conceptually difficult, because the problem lacks a small parameter, and therefore, the perturbative expansions in powers of the disorder potential diverge. At the single-particle level, the problem has been solved by Wegner [32], who found an exact expression for the single-particle propagator. The Wegner propagator does not reveal any singularities and describes a state of the matter without pronounced resonances and consequently without clearly defined quasiparticles. However, the difficulties aggregate by far if one goes beyond the single-particle picture and considers processes involving two or more particles.

Our main intention is to calculate the static conductivity of disordered graphene in a strong magnetic field. In the lowest Landau level, the spectrum of the gapless and chemically neutral graphene Hamiltonian has zero energy, which are the consequence of its band topology and responsible for the exceptional transport properties under normal conditions. The spectral gap or fluctuations of the Fermi energy due to the hopping between second-nearest neighbors on the honeycomb lattice split this zero mode in two sublevels. Surprisingly, the static conductivity of the clean system evaluated from the Kubo–Greenwood formula gives a conductivity within infinitely thin parametric windows around the zero mode. One would expect that the disorder broadens this window to considerable sizes. However, because the perturbative series for the two-particles propagator diverges, a naive use of the Kubo formula fails. Therefore, we approach the conductivity via the Einstein relation, which requires the knowledge of the density of states and of the diffusion coefficient. While the former is known from the Wegner’s solution, the latter is not. To deal with such divergences, we develop an analytical approach based on a self-consistent equation for the mean square displacement, which allows one to directly extract the diffusion coefficient and static conductivity.

Following the line of Wegner’s exact considerations, we determine the general expression of the density of states of graphene. With a gap, the density of states of weakly disordered graphene represents two sharp peaks centered around each of the sublevels. For the case of gapless and chemically neutral graphene, both peaks coalesce to a single one with twice the height. The diffusion coefficient is extracted from the time evolution of the mean squared displacement. The latter tends toward a stationary state, which would reestablish the situation we observe in the clean system with all electrons distributed between stationary orbits. However, our findings suggest an infinitely large time needed for the system to arrive in this state. At the intermediary time scales, the mean squared displacement behaves lineary in time from which the diffusion coefficient is extracted. The combination of the density of states and diffusion coefficient, known as the Einstein relation, gives a universal, i.e., disorder independent value for the static conductivity. At the band center of the lowest Landau level, we find for the conductivity a universal value ∼0.72 e${}^{2}$/h, which is surprisingly close to the established results for the conductivity of the disordered Dirac electrons. In the subsequent work, we intend to extend our analysis to higher Landau levels and to address the Hall conductivity with the aim of arriving at an effective description of a kind of the Chern–Simons theories.

The quantum Hall effect has long become the standard tool for high-precision measurements and adjustments [58]. With our clearly laid out prediction for the dc conductivity in the lowest Landau level of graphene, we have provided a benchmark for prospective graphene-based metrological standardization devices.

## Data Availability

The data presented in this study are available on request from the corresponding author.

## References

[1] Abrahams E., Anderson P.W., Licciardello D.C., Ramakrishnan T.V. (1979). Scaling Theory of Localization: Absence of Quantum Diffusion in Two Dimensions. Phys. Rev. Lett..

[2] Gor’kov L.G., Larkin A.I., Khmel’nitskii D.E. (1979). Particle conductivity in a two-dimensional random potential. JETP Lett..

[3] Hikami S., Larkin A., Nagaoka Y. (1980). Spin-Orbit Interaction and Magnetoresistance in the Two Dimensional Random System. Prog. Theor. Phys..

[4] Vollhardt D., Wölfle P. (1980). Diagrammatic, self-consistent treatment of the Anderson localization problem in *d*⩽2 dimensions. Phys. Rev. B.

[5] Hanein Y., Meirav U., Shahar D., Li C.C., Tsui D.C., Shtrikman H. (1998). The metallic like conductivity of a two-dimensional hole system. Phys. Rev. Lett..

[6] Novoselov K.S., Geim A.K., Morozov S.V., Jiang D., Katsnelson M.I., Grigorieva I.V., Dubonos S.V., Firsov A.A. (2005). Two-dimensional gas of massless Dirac fermions in graphene. Nature.

[7] Tan Y.-W., Zhang Y., Bolotin K., Zhao Y., Adam S., Hwang E.H., Das Sarma S., Stormer H.L., Kim P. (2007). Measurement of scattering rate and minimal conductivity in graphene. Phys. Rev. Lett..

[8] Elias D.C., Nair R.R., Mohiuddin T.M.G., Morozov S.V., Blake P., Halsall M.P., Ferrari A.C., Boukhvalov D.W., Katsnelson M.I., Geim A.K. (2009). Control of graphene’s properties by reversible hydrogenation: Evidence for graphane. Science.

[9] Allen M.J., Tung V.C., Kaner R.B. (2010). Honeycomb carbon: A review of graphene. Chem. Rev..

[10] Chen L., Liu C.-C., Feng B., He X., Cheng P., Ding Z., Meng S., Yao Y., Wu K. (2012). Evidence for Dirac fermions in a honeycomb lattice based on silicon. Phys. Rev. Lett..

[11] Castro Neto A.H., Guinea F., Peres N.M.R., Novoselov K.S., Geim A.K. (2009). The electronic properties of graphene. Rev. Mod. Phys..

[12] Kotov V.N., Uchoa B., Pereira V.M., Guinea F., Castro Neto A.H. (2012). Electron-Electron Interactions in Graphene: Current Status and Perspectives. Rev. Mod. Phys..

[13] Hasan M.Z., Kane C.L. (2010). Colloquium: Topological insulators. Rev. Mod. Phys..

[14] Qi X.-L., Zhang S.-C. (2011). Topological insulators and superconductors. Rev. Mod. Phys..

[15] Avsar A., Ochoa H., Guinea F., Özyilmaz B., Van Wees B.J., Vera-Marun I.J. (2020). Colloquium: Spintronics in graphene and other two-dimensional materials. Rev. Mod. Phys..

[16] Bernevig B.A., Hughes T.L., Zhang S.-C. (2006). Quantum spin Hall effect and topological phase transition in HgTe quantum wells. Science.

[17] Shon N.H., Ando T. (1998). Quantum transport in two-dimensional graphite system. J. Phys. Soc. Jpn..

[18] Ando T., Zheng Y., Suzuura H. (2002). Dynamical conductivity and zero-mode anomaly in honeycomb lattices. J. Phys. Soc. Jpn..

[19] Suzuura H., Ando T. (2002). Crossover from symplectic to orthogonal class in a two-dimensional honeycomb lattice. Phys. Rev. Lett..

[20] McCann E., Kechedzhi K., Fal’ko V.I., Suzuura H., Ando T., Altshuler B.L. (2006). Weak-localization magnetoresistance and valley symmetry in graphene. Phys. Rev. Lett..

[21] Altshuler B.L., Aronov A.G., Larkin A.I., Khmel’nitskii D.E. (1981). Anomalous magnetoresistance in semiconductors. Sov. Phys. JETP.

[22] Altshuler B.L., Simons B.D., Akkermans E., Montambaux G., Pichard J.-L., Zinn-Justin J. (1995). Universalities: From Anderson localization to quantum chaos. Mesoscopic Quantum Physics, Les Houches 1994.

[23] Efetov K. (1997). Supersymmetry in Disorder and Chaos.

[24] Lee P.A. (1993). Localized states in a d-wave superconductor. Phys. Rev. Lett..

[25] Wegner F.J. (1979). The mobility edge problem: Continuous symmetry and a conjecture. Z. Physik B.

[26] Schäfer L., Wegner F.J. (1980). Disordered system withn orbitals per site: Lagrange formulation, hyperbolic symmetry, and Goldstone modes. Z. Physik B.

[27] Hikami S. (1981). Anderson localization in a nonlinear-*σ*-model representation. Phys. Rev. B.

[28] Wegner F.J. (1979). Disordered system with n orbitals per site: *n*=*∞* limit. Phys. Rev. B.

[29] McKane A.J., Stone M. (1981). Localization as an alternative to Goldstone’s theorem. Ann. Phys..

[30] Fradkin E. (1986). Critical behavior of disordered degenerate semiconductors. II. Spectrum and transport properties in mean-field theory. Phys. Rev. B.

[31] Ando T. (1974). Theory of quantum transport in a two-dimensional electron system under magnetic field. III. Many-site approximation. J. Phys. Soc. Jpn..

[32] Wegner F.J. (1983). Exact density of states for lowest Landau level in white noise potential. Superfield representation for interacting systems. Z. Phys. B Condens. Matter.

[33] Brézin E., Gross D.J., Itzykson C. (1984). Density of states in the presence of a strong magnetic field and random impurities. Nucl. Phys. B.

[34] Hikami S. (1984). Borel-Padé analysis for the two-dimensional electron in a random potential under a strong magnetic field. Phys. Rev. B.

[35] Hikami S. (1984). Anderson Localization of the two-dimensional electron in a random potential under a strong magnetic field. Prog. Theor. Phys..

[36] Aoki H. (1987). Quantised Hall effect. Rep. Prog. Phys..

[37] Tkachov G. (2015). Topological Insulators: The Physics of Spin Helicity in Quantum Transport.

[38] Sinner A., Ziegler K. (2014). Two-parameter scaling theory of transport near a spectral node. Phys. Rev. B.

[39] Sinner A., Ziegler K. (2016). Finite-size scaling in a 2D disordered electron gas with spectral nodes. J. Phys. Condens. Matter.

[40] Goldenfeld N. (1992). Lectures on Phase Transitions and the Renormalization Group.

[41] Huang K. (1987). Statistical Mechanics.

[42] Chaikin P.M., Lubenski T.C. (1995). Principles of Condensed Matter Physics.

[43] Ziegler K. (2012). Quantum diffusion in two-dimensional random systems with particle–hole symmetry. J. Phys. A Math. Theor..

[44] Singh R.R.P., Chakravarty S. (1986). A disordered two-dimensional system in a magnetic field: Borel-Padé analysis. Nucl. Phys. B.

[45] Hikami S., Shirai M., Wegner F.J. (1993). Anderson localization in the lowest Landau level for a two-subband model. Nucl. Phys. B.

[46] Culcer D., Keser A.C., Li Y., Tkachov G. (2020). Transport in two-dimensional topological materials: Recent developments in experiment and theory. 2D Mater..

[47] König M., Buhmann H., Molenkamp L.W., Hughes T., Liu C.-X., Qi X.-L., Zhang S.-C. (2008). The quantum spin Hall effect: Theory and experiment. J. Phys. Soc. Jpn..

[48] Li G., Andrei E.Y. (2007). Observation of Landau levels of Dirac fermions in graphite. Nat. Phys..

[49] Goswami P., Jia X., Chakravarty S. (2007). Quantum Hall plateau transition in the lowest Landau level of disordered graphene. Phys. Rev. B.

[50] Ludwig A.W.W., Fisher M.P.A., Shankar R., Grinstein G. (1994). Integer quantum Hall transition: An alternative approach and exact results. Phys. Rev. B.

[51] Ziegler K. (2006). Robust transport properties in graphene. Phys. Rev. Lett..

[52] Ziegler K. (2007). Minimal conductivity of graphene: Nonuniversal values from the Kubo formula. Phys. Rev. B.

[53] Sinner A., Ziegler K. (2018). Conductivity of disordered 2d binodal Dirac electron gas: Effect of internode scattering. Philos. Mag..

[54] Sinner A., Tkachov G. (2022). Diffusive transport in the lowest Landau level of disordered 2d semimetals: The mean-square-displacement approach. Eur. Phys. J. B.

[55] Novoselov K.S., Jiang Z., Zhang Y., Morozov S.V., Stormer H.L., Zeitler U., Maan J.C., Boebinger G.S., Kim P., Geim A.K. (2007). Room-temperature quantum Hall effect in graphene. Science.

[56] Jiang Z., Zhang Y., Tan Y.-W., Stormer H.L., Kim P. (2007). Quantum Hall effect in graphene. Solid State Comm..

[57] Shemer Z., Barkai E. (2009). Einstein relation and effective temperature for systems with quenched disorder. Phys. Rev. E.

[58] Jeckelmann B., Jeanneret B. (2001). The quantum Hall effect as an electrical resistance standard. Rep. Prog. Phys..

